# New Insights into the Evolution of Metazoan Tyrosinase Gene Family

**DOI:** 10.1371/journal.pone.0035731

**Published:** 2012-04-20

**Authors:** Rosaria Esposito, Salvatore D'Aniello, Paola Squarzoni, Maria Rosa Pezzotti, Filomena Ristoratore, Antonietta Spagnuolo

**Affiliations:** Cellular and Developmental Biology Department, Stazione Zoologica Anton Dohrn, Villa Comunale, Napoli, Italy; Laboratoire Arago, France

## Abstract

Tyrosinases, widely distributed among animals, plants and fungi, are involved in the biosynthesis of melanin, a pigment that has been exploited, in the course of evolution, to serve different functions. We conducted a deep evolutionary analysis of tyrosinase family amongst metazoa, thanks to the availability of new sequenced genomes, assessing that tyrosinases (tyr) represent a distinctive feature of all the organisms included in our study and, interestingly, they show an independent expansion in most of the analyzed phyla. Tyrosinase-related proteins (tyrp), which derive from tyr but show distinct key residues in the catalytic domain, constitute an invention of chordate lineage. In addition we here reported a detailed study of the expression territories of the ascidian *Ciona intestinalis tyr* and *tyrps*. Furthermore, we put efforts in the identification of the regulatory sequences responsible for their expression in pigment cell lineage. Collectively, the results reported here enlarge our knowledge about the *tyrosinase* gene family as valuable resource for understanding the genetic components involved in pigment cells evolution and development.

## Introduction

In vertebrates three types of melanin-producing pigment cells are known, that have distinct, even if related, embryonic origins: melanocytes of the inner ear, skin, hair-bulbs and uvea, which derive from the neural crest; retinal pigment epithelium (RPE) cells of the eye derived from the neural tube; and pigment cells of the pineal organ, which also arise from the neural tube [1], [2], [3]. All these cells share the capacity to produce melanins, a class of polymeric pigments whose biosynthesis is mainly governed by evolutionarily conserved enzymes of the tyrosinase family: tyrosinase (tyr), tyrosinase related protein-1 (tyrp1) and tyrosinase related protein-2 (tyrp2) also called DOPAchrome tautomerase (dct). Amongst them, tyr plays the initial and crucial role for melanin production, by converting the amino acid tyrosine to 3,4-dihydroxyphenylalanine (DOPA), while tyrp1 and tyrp2 function in subsequent steps, since they influence the quantity and the quality of the synthesized melanins [4], [5]. Furthermore, both tyrps are known to stabilize the tyr enzyme [6], [7], [8] and to function in melanocyte survival and maintenance of melanosomal structures [9].

The genetic programs leading to the development of the three types of vertebrate pigment cells, although different, thus converge at a certain point to allow the expression of members of the *tyrosinase* family, in order to produce melanin pigments. It is noteworthy that many human genetic inheritable pathologies, as multiple forms of albinism, vitiligo and deafness, are linked to genetic mutations in one or more genes responsible for melanin biosynthesis [10]. These genes therefore represent a good paradigm to answer questions regarding the evolution, genetics, and developmental biology of pigment cells, as well as to approach human disorders associated with defects in their synthesis, regulation or function.

The three tyrosinase family proteins, besides showing extensive similarities at the amino acid level, share many key structural characteristics (see [8], [11] for detailed reviews). The first one consists of the presence of two highly conserved metal binding domains, MeA and MeB, that are involved in the proper folding of the active site and in the binding of metal cofactors (copper for tyr, zinc for tyrp2 and unknown for tyrp1). Few differences exist, consisting in four amino acid substitutions, which might be responsible for the switch of affinity from phenolic substrates, typical of tyr enzymes, to indolic substrates, observed in tyrps. A further interesting common trait of tyr and tyrps is the presence of three cysteine clusters, two at the N-terminal and one located between MeA and MeB, likely involved in correct protein folding [8].

Data collected so far have suggested that the *tyr* and *tyrp* gene family has clearly evolved from a common ancestral *tyrosinase* gene [12], [13] that was first duplicated before the divergence of urochordates (ascidians) and vertebrates [14], leading to *tyrosinase* (*tyr*) and a *tyrosinase-related protein* (*tyrp*). The *tyrp* was then duplicated early in vertebrate lineage, before the divergence of teleost fishes [15], giving rise to *tyrp1* and *tyrp2* (or *dct*).

However, a survey of the protochordate ascidian *Ciona intestinalis* genome revealed the presence of three *tyrosinase* family genes, one *tyr* (*Ci-tyr*) and two *tyrps* (*Ci-tyrp1/2a* and *Ci-tyrp1/2b*) [16], thus indicating that *tyr* family evolution might be much more complex than previously thought. As a model system for understanding chordate development, ascidians, such as *C. intestinalis*, offers important experimental advantages, compared to vertebrate species. They produce a large number of embryos, have external development, are small in size and have a fixed cell lineage. Furthermore, they have two pigmented sensory organs in the sensory vesicle: the otolith, composed of one pigmented cup cell, which functions in geotactic responses, and the ocellus, involved in photoreception, which is composed of three lens cells, 30 photoreceptor cells and one pigment cell [17]. It is noteworthy that the cell-lineage of the pigment cells has been fully documented [18]; furthermore in ascidians every blastomere of the embryo is distinguishable, so that it is easy to precisely identify cells expressing genes of interest, when gene expression is initiated and lineage in which gene expression is inherited [19]. This peculiarity coupled with the possibility, in *C. intestinalis*, to easily isolate the promoter regions of the gene of interest, by using electroporation of chimeric reporter genes [20], makes *Ciona* a model system ideal to identify marker genes, specific for each lineage, and study the genetic cascades in which they are involved.

In the present study, as a first approach, we have exploited the growing number of sequenced genomes, from different taxa, for a deeper evolutionary analysis in order to shed light on the origin of *tyrosinase* family genes. We have then devoted our attention to the *C. intestinalis tyrosinase* family members, by conducting a detailed characterization of the expression profiles of the two *Ci-tyrps*, in comparison with *Ci-tyr*. Furthermore, analyses of their transcriptional regulation led to the identification of regulatory regions responsible for their spatio-temporal expression during *Ciona* embryogenesis. These enhancers have been successfully used as tools to study the genetic circuits controlling pigment cell differentiation during *Ciona* embryogenesis [21]. These enhancers will be also instrumental to look for modules responsible for the expression patterns of *tyrosinase* family genes in *Ciona*.

## Results

### Tyrosinase family evolution

To study the evolutionary history of tyrosinase family we conducted a phylogenetic analysis by using deduced protein sequences from eumetazoan available genomes. Among bilaterians we included sequences from deuterostomes, as vertebrates, urochordates (*C. intestinalis* and *Ciona savignyi*) [22], [23], cephalocordates (*Branchiostoma floridae*) [24], hemichordates (*Saccoglossus kowalevskii*), and from protostomes, as nematodes (*Caenorhabditis elegans*) [25] and molluscs (*Sepia officinalis, Loligo vulgaris, Pinctada fucata*). Among radiates, tyrosinases from cnidarian genomes (*Nematostella vectensis* and *Hydra magnipapillata*) [26] were also included, while no ctenophore's representatives were found. A tyrosinase-like sequence from sponges (*Suberites domuncula*), which are historically considered to be the earliest diverging metazoan phylum, was used as outgroup. We were unable to identify any putative sequence related to the tyrosinase family in available echinoderm, annelid and arthropod genomes. It is already known that arthropods use phenoloxidases, enzymes that belong, as tyrosinases, to the Type3 Copper protein family, for melanin biosynthesis [27], and there are evidences indicating that also annelids and echinoderms could exploit phenoloxidases, in place of tyrosinases, for this cellular process [28], [29].

**Figure 1:**
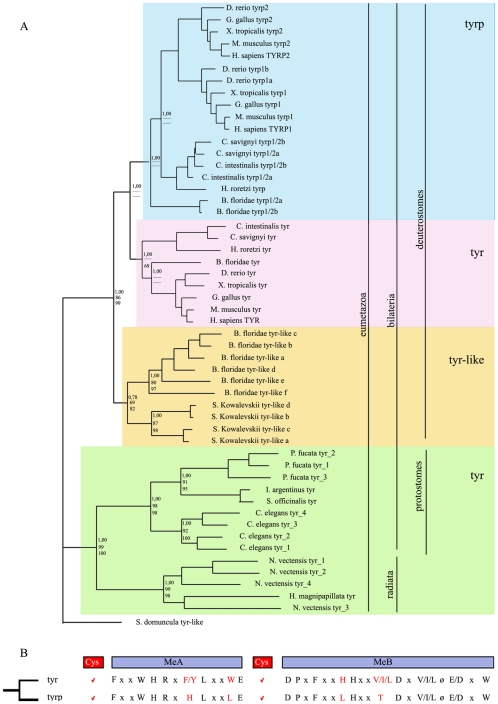
Evolution of tyr and tyrps in metazoa. A) Phylogenetic study of tyrosinase family proteins. Numbers at the branches indicate bootstrap values obtained with MrBayes, Maximum Likelihood and Neighbour Joining methods, respectively. Vertical bars highlight the classification of the analysed eumetazoa in radiata and bilateria, which are in turn subdivided in protostomes and deuterostomes. Colored boxes highlight four groups of proteins: cnidarian and protostome tyrosinases (green box), cephalochordate and hemichordate tyrs-like (orange box), chordate “canonical” tyrosinases (red box), and chordate tyrps (blue box). B) Conserved aminoacid residues in the two metal binding domains (MeA and MeB) of tyrosinase family members, derived from a multiple sequence alignment (see Fig. S2). Key aminoacid positions, probably involved in the change of affinity for phenolic substrates, are reported in red. ø indicates aromatic residues (F, Y or W), x indicates any aminoacid. The position of cysteine clusters (red boxes) refers to deuterostomes (see also Fig. S3).

The topology of our phylogenetic reconstruction revealed the clustering of four distinct groups of proteins (Fig. 1A): 1. cnidarian and protostome tyrs (green box), 2. chordate “canonical” tyrs (pink box), 3. chordate tyrps (blue box) and 4. a group of tyrs, present in cephalochordates and hemichordates, that branched independently and that we called tyrs-like (orange box), given the lack of any functional information. In this phylogenetic reconstruction the cnidarian tyrs grouped with protostome tyrs and not at the base of bilaterian tyrs, as it could be expected from phylogenetic lineage relationships. Notably, tyr and tyr-like independent expansions were observed in most of the analyzed metazoan phyla (Fig. 1A), whose functional significance is still unknown, thus opening the evolutionary history of this gene family to new perspectives.

In order to gain insight into evolutionary phylogenesis of the expanded tyrosinases, we analyzed, when available, the chromosomal distribution of all the *tyrosinase* expanded genes in chordates (*B. floridae*), hemichordates (*S. kowalevskii*), nematodes (*C. elegans*) and cnidarians (*N. vectensis*). The data showed that only *S. kowalevskii* and *C. elegans* expanded *tyrosinases* are contained in pair on two scaffolds or chromosomes (Fig. S1) and this indicates tandem duplication events, but we cannot exclude that future chromosomal reconstructions in other genome models would give a similar layout.

**Figure 2:**
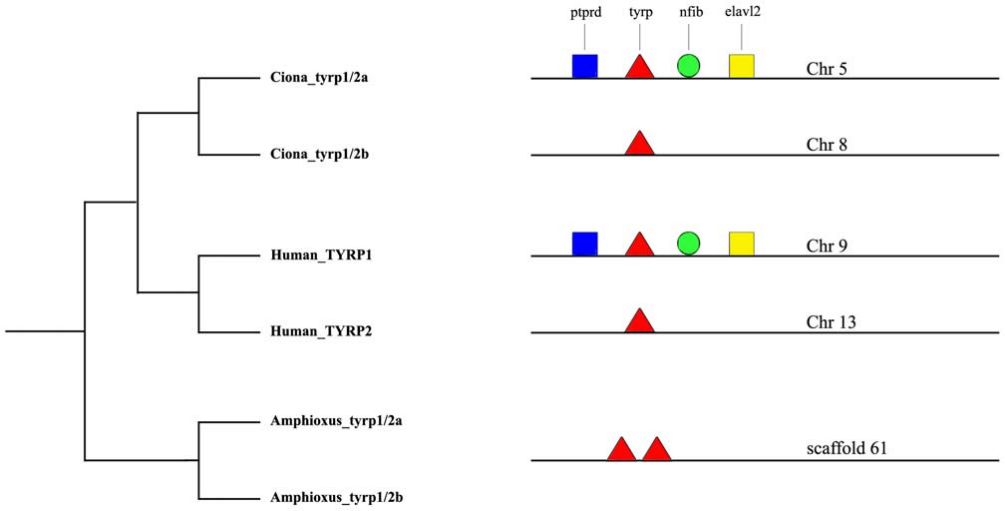
Tyrosinase-related proteins evolution in chordates. Schematic representation of synteny analysis of tyrosinase-related proteins in Ciona (*C. intestinalis*), amphioxus (*B. floridae*) and human (*H. sapiens*). Tyrps are represented by the red triangle. Our analysis revealed syntenic conservation shared by *Ci-tyrp1/2a* on chromosome 5 and *Hs-TYRP1* on chromosome 9. Neighbouring genes *ptprd* (blue square), *nfIB* (green circle) and *elavl2* (yellow square) are indicated.

The present survey assessed that tyrosinase-related proteins (tyrp) are present exclusively in chordates; however ascidian and cephalochordate tyrps showed no clear phylogenetic relationships with vertebrate tyrp1 and tyrp2. In an effort to gain more insights into the evolutionary history of these genes, we thus studied the tyrp synteny conservation in amphioxus, ascidian and human genomes and we mapped three independent gene duplications. In amphioxus *tyrp1/2a* and *tyrp1/2b* came from a tandem duplication event, since they lay close on scaffold 61, but we could not establish any synteny conservation with *Ciona* and human *tyrps*, possibly due to the short length of the scaffold (Fig. 2). On the other hand, we detected synteny conservation for *Ci-tyrp1/2a*, on chromosome 5, and human *TYRP1*, on chromosome 9, indicating that these genes are clearly orthologous. No shared genes around the locus of *Ci-tyrp1/2b*, on chromosome 8, and human *TYRP2*, on chromosome 13, were instead identified (Fig. 2), so we could not infer or exclude any orthology in this case.

A detailed analysis of conserved metal binding domains (MeA and MeB), based on previous work [8], was conducted on all tyrosinase family members included in our study. We confirmed that few key residues within the metal binding domains (MeA and MeB) are clearly archetypal of tyr or tyrp proteins [8]. These residues thus represent an important tool to easily distinguish between tyr and tyrps and allocate family memberships (Fig. 1B and Fig. S2). These residues were instrumental, in our analysis, to assign protostome and cnidarian sequences to the tyrosinase group in support of our phylogenetic tree (Fig. 1B and Fig. S2).

A further known characteristic of *tyrosinase* gene family is the presence of cysteine clusters that are probably responsible for correct protein folding. We detected an high degree of cysteine conservation, both at the N-terminal and between MeA and MeB, in the deuterostome proteins (Fig. S3). In the protostome lineage the cysteine clusters appeared conserved at the N-terminal, although with a lower number of cysteine residues; no cysteine cluster was detected between MeA and MeB domains whereas, interestingly, a specific cluster was present at the C-terminus in both nematodes and molluscs (Fig. S3).

### Expression pattern of *tyrosinase* family genes in *Ciona intestinalis*

Expression patterns of *Ci-tyr*, *Ci-tyrp1/2a* and *Ci-tyrp1/2b* were examined through whole mount *in situ* hybridization experiments on *Ciona* embryos at different developmental stages. No signal was detected up to the late gastrula stage.

**Figure 3:**
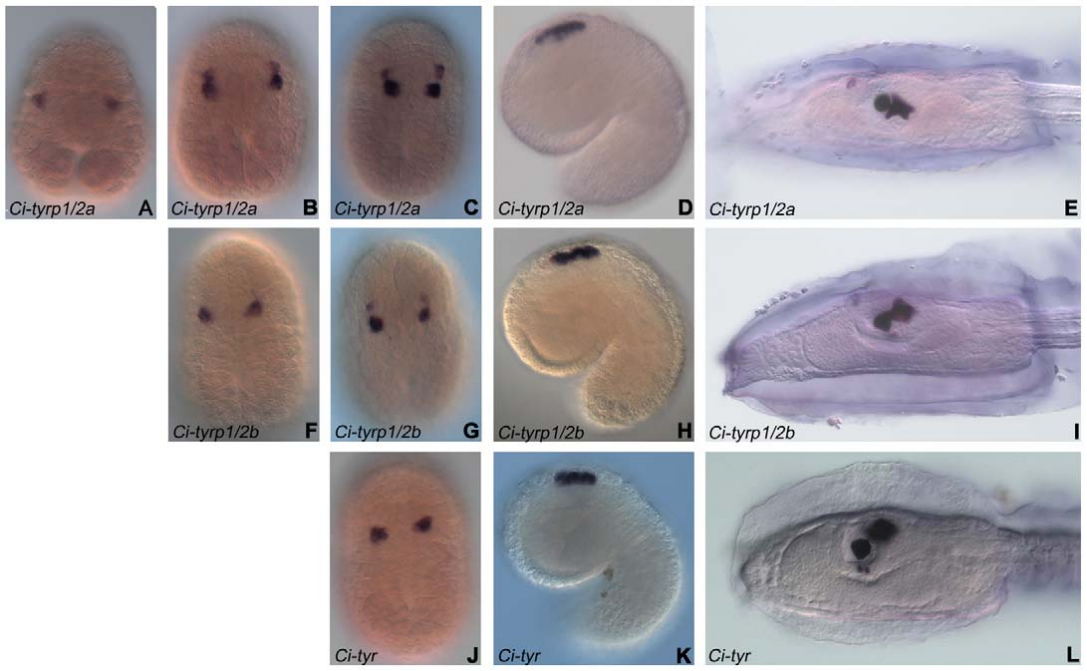
*Ci-tyr-tyrps* expression in pigment cell precursors. Expression pattern of *Ci-tyrp1*/2a (A–E), *Ci-tyrp1/2b* (F–I) and *Ci-tyr* (J–L) in *C. intestinalis* embryos at different developmental stages, detected through whole mount *in situ* hybridization experiments. The three genes are specifically expressed in a9.49 blastomeres (pigment cell precursors) and in their descendant cells from late gastrula (*Ci-tyrp1/2a*, in A), middle neurula (*Ci-tyrp1/2b*, in F) or late neurula (*Ci-tyr*, in J) stages up to the larval stage (E, I, L). A: late gastrula stage, vegetal view. B, F: middle neurula stage, dorsal view. C, G, J: late neurula stage, dorsal view. D, H, K: mid-tailbud stage, lateral view. E, I, L: larval stage, lateral view. A, B, C, F, G, J: anterior is up; D, E, H, I, K, L: anterior is on the left.

*Ci-tyrp1/2a* was the first to be expressed, from the late gastrula stage, in the a9.49 blastomere pair which corresponds to the pigment cell precursors (Fig. 3A). A clear and specific signal was then inherited in both a9.49 progeny (the a10.97 and a10.98 pairs) appearing much stronger in the posterior a10.97, compared to the anterior a10.98 pairs, at middle and late neurula stages (Figs. 3B and 3C). The expression persisted, with the same intensity up to the tailbud stage, in these four blastomeres that line up along the dorsal midline of the developing neural tube (Fig. 3D). The posterior a10.97 cells then differentiate into the otolith and ocellus pigment cells, where the *Ci-tyrp1/2a* mRNA remained localized at the larval stage (Fig. 3E).

*Ci-tyr* and *Ci-tyrp1/2b* expression territories were superimposable with that of *Ci-tyrp1/2a*. The only difference was that their hybridization signals were first detected at a slightly delayed developmental time, the middle (*Ci-tyrp1/2b*, Figs. 3F, 3G, 3H, 3I) and late (*Ci-tyr*, Figs. 3J, 3K, 3L) neurula stages, compared to *Ci-tyrp1/2a*. These results confirm previous data on *tyrosinase* expression in *C. intestinalis* [30] and strengthen the evidence that *tyrosinase* family members are specific markers of *C. intestinalis* pigment cell lineage from the late gastrula stage.

### *In vivo cis*-regulatory regions analysis

**Figure 4:**
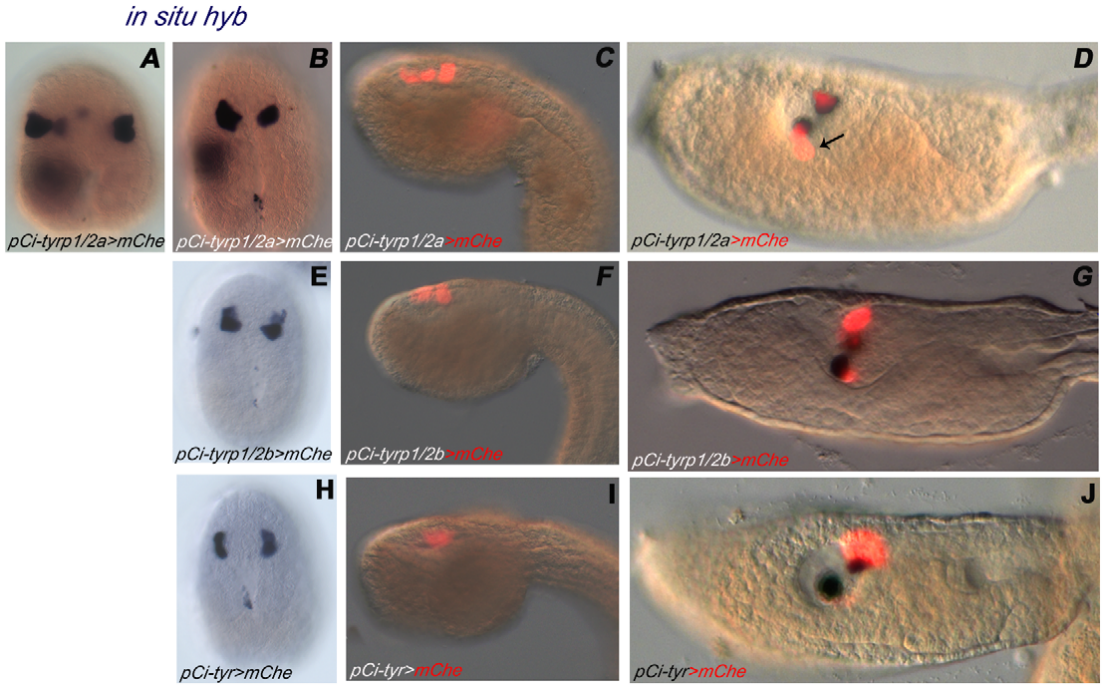
Identification of pigment cell *Ci-tyr* and *tyrps* enhancers. *In vivo* analysis of *pCi-tyrp1/2a>mChe* (A–D), *pCi-tyrp1/2b>mChe* (E–G) and *pCi-tyr>mChe* (H–J) constructs performed through transgenesis *via* electroporation experiments. Merged bright- field/fluorescent images of mCherry expression driven by the three enhancers at the tailbud (C,F,I) and larval stages (D,G,J). Lateral view, anterior is on the left. Note transgene expression in the pigment cell lineage. Arrow in D indicates the accessory cell in which a fluorescent signal is present. *In situ* hybridization experiments, using mCherry antisense RNA as probe, were performed in order to reveal the reporter expression at earlier developmental stages, when fluorescence is still undetectable (A: late gastrula stage; B, E, H: neurula stage, dorsal view). Reporter gene expression perfectly mirrors endogenous genes expression profiles (compare with Fig. 3).

To test the transcription driving activity of *Ci-tyr*, *Ci-tyrp1/2a* and *Ci-tyrp1/2b*, the 5′ genomic regions of each gene were isolated by PCR on *C. intestinalis* genomic DNA. Each upstream fragment was mapped, between the ATG of the transcript and the contiguous 5′ gene, and the length corresponded to 0.9 kb for *pCi-tyr*, and 1.5 kb for both *pCi-tyrp1/2a* and *pCi-tyrp1/2b*. These putative promoters were cloned upstream of a *mCherry* reporter gene (constructs *pCi-tyr>mChe pCi-tyrp1/2a>mChe*, and *pCi-tyrp1/2b>mChe*) and tested, by transgenesis *via* electroporation, for the capability to direct pigment cell lineage-specific expression of the reporter at the larval stage. The data indicated that the *pCi-tyr*, *pCi-tyrp1/2a* and *pCi-tyrp1/2b* all behave like specific enhancers, since the larvae showed a fluorescent signal in the otolith and/or ocellus pigment cells in a high proportion of the electroporated embryos (80–85% for *pCi-tyrp1/2a>mChe*, 60% for *pCi-tyr>mChe* and 50% for *pCi-tyrp1/2b>mChe* constructs) (Figs. 4D, 4G and 4J). The *pCi-tyrp1/2a* appeared the strongest, since many larvae showed a robust signal in the two pigment cells and, in a lower percentage, also in one or two accessory cells in the brain vesicle, that could represent the a10.97 sister cells, the a 10.98 pair, given the long half-life of mCherry protein (Fig. 4D). Furthermore, *pCi-tyrp1/2a* activity was the first to be detected as fluorescent protein product from early tailbud stage (data not shown), compared to the late tailbud (Fig. 4C) stage when mCherry protein signal, driven by *pCi-tyr* or *pCi-tyrp1/2b*, started to appear (Figs. 4F and 4I), confirming the timing of the *in situ* hybridization signal. To check for reporter expression at earlier developmental stages, whole mount *in situ* hybridization experiments, using *mCherry* antisense RNA, were performed on embryos electroporated with *pCi-tyr>mChe*, *pCi-tyrp1/2a>mChe*, and *pCi-tyrp1/2b>mChe* constructs, at late gastrula and neurula stages. The presence of *mCherry* mRNA in the territories where the endogenous genes are expressed (Figs. 4A, 4B, 4E and 4H compared with the Fig. 3) confirmed that the three promoters we have isolated contain the *cis*-regulatory information required for a correct spatial and temporal expression of the corresponding genes.

Comparisons between orthologous *C. intestinalis* and *C. savignyi* sequences have already indicated that these two species are at sufficient evolutionary distance to permit efficient identification of conserved regulatory sequence information [21], [31], [32]. Phylogenetic footprinting of the three corresponding promoters, *pCi-tyr*, *pCi-tyrp1/2a* and *pCi-tyrp1/2b*, pointed to the presence of a 400 bp highly conserved fragment only in the *pCi-tyrp1/2a* 5′ regulatory sequence. A 600 bp region, including this fragment and extending up to the ATG of *Ci-tyrp1/2a*, named *pCi-tyrp1/2a-0.6*, was cloned upstream of *mCherry* reporter and tested *in vivo* by transgenesis through electroporation. The results indicated that *pCi-tyrp1/2a-0.6* had similar activity to *pCi-tyrp1/2a* (Fig. S4), thus permitting an initial dissection of the regulatory region directing *Ci-tyrp1/2a* transcription.

This *pCi-tyrp1/2a-0.6* fragment was then subjected to bioinformatic analyses, in comparison with the corresponding region of *C. savignyi*. This initial approach was mostly focused on the search for consensus motifs, conserved between *Ci-tyrp1/2a* and *Cs-tyrp1/2a* corresponding enhancers, for representatives of families already demonstrated, in vertebrates, to act as important players in pigmentation processes, such as Pax, Oct/Pou, Sox-TCF (HMG family), Mitf-TFE (bHLH-LZ family binding E-box motif) (for a comprehensive review see [33], [34]). In this analysis we also included the promoter fragment *Hr-tyrp-333N*, previously identified in the ascidian *Halocynthia roretzi*, and demonstrated to be sufficient for *Hr-tyrp* expression in pigment cell precursors [35].

**Figure 5:**
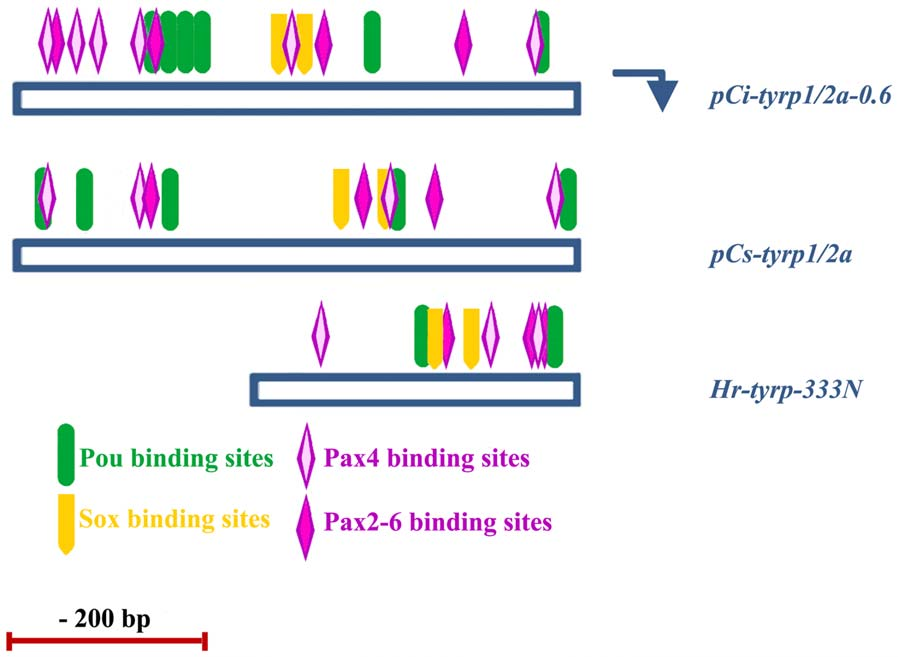
Putative transcription factors binding sites on *pCi-tyrp1/2a-0.6*, *pCs-tyrp1/2a* and *Hr-tyrp-333N* fragments. Bars indicate the analyzed *pCi-tyrp1/2a-0.6*, *pCs-tyrp1/2a* and *Hr-tyrp-333N* fragments. Transcription starting site is indicated by the blue arrow. Putative binding sites for Pou, Pax4, Pax 2–6 and Sox are taken into consideration and respectively represented by colored shapes, as indicated on the bottom.

The software we used for this analysis (http://alggen.lsi.upc.es/cgi-bin/promo_v3/promo/promoinit.cgi?dirDB=TF_8.3) identified different putative binding sites for Pax, Oct/Pou and Sox family genes. Interestingly, no canonical Mitf binding sites were identified in these regions. The study revealed also that Pax, Oct/Pou and Sox binding sites were organized in modules that are well conserved between *C. intestinalis* and *C. savignyi* and partially conserved also with *H. roretzi* (Fig. 5).

## Discussion

Melanins are formed *in vitro* from L-tyrosine in the presence of tyrosinase alone; this led to the deduction that melanogenesis is a simple process requiring a single enzyme, the tyrosinase. This is the case in bacteria, sponges and plants; in the course of animal evolution, however, the situation has become more and more complex up to the mammals where the process is very sophisticated and has to be tightly regulated in terms of amount, type of melanin produced and the environment in which the synthesis takes place. Thus we move from a simple to a complex multi-enzymatic process (as in mammalian melanocytes), where new family members, the tyrps, have been added to finely tune the whole pathway. Sequence comparison of tyr and tyrps reveals that these proteins share many key structural features, indicating their common origin from an ancestral *tyrosinase* gene able to catalyse the critical rate-limiting hydroxylation of L-tyrosine to L-DOPA [11].

### Melanogenic toolkit in animal evolution

Our phylogenetic analysis led to the conclusion that the tyrosinase family is divided in four distinct branches: 1. cnidarian and protostome tyrs (green box), 2. cephalochordate and hemichordate tyrs-like (orange box), 3. chordate “canonical” tyrs (pink box) and 4. chordate tyrps (blue box) (Fig. 1A).

The most parsimonious evolutionary scenario, based on the available data of sequenced genomes and gene predictions, as well as taking into account insights from enzymatic activity studies, is that one tyrosinase was present in the ancestor of eumetazoa. Indeed protostomes and radiates possess only one representative *tyr* gene that, in some cases, has been subjected to a lineage specific expansions (green box, Fig. 1A).

In the ancestor of deuterostomes then occurred a duplication that produced two *tyrosinase* genes. One of them was lost in tunicates and vertebrates and was retained only by cephalochordates and hemichordates. We named this group of genes as *tyr-like* (pink box), since no functional assays attesting their tyrosinase activity are yet available. The second *tyrosinase* gene was lost in hemichordates and was further amplified in the ancestor of chordates, giving rise to the canonical tyr (blue box) and tyrps (orange box).

An alternative evolutionary hypothesis is that two tyrosinase genes existed in the ancestor of metazoa. This scenario would imply that one representative (gene 1) has been retained in deuterostomes and lost in protostomes and radiates, while the second representative (gene 2) has been lost in deuterostomes and retained in protostomes and radiates. Thought we do not have extensive data to support either one of the two hypotheses, novel sequencing data, from animal taxa in key positions in the three of life, will help clarifying the phylogenetic history of this gene family.

We were unable to identify any putative sequence related to the *tyrosinase* family in available arthropod, echinoderm, and annelid genomes. Previous studies have demonstrated that arthropods use the phenoloxidases for melanin byosinthesis, in place of tyrosinases [27] and that in these organisms the melanin, apart from providing pigmentation, is involved in other important processes, as wound healing, sclerotization and immunity [36], [37]. It is intriguing to note that there are evidences indicating that annelids and echinoderms use phenoloxidases for both melanin byosinthesis and immune defence [28], [29], thus supporting the absence of proteins phylogenetically related to tyrosinases in these organisms.

Our study put in light also a novel and intriguing example of independent family expansion in metazoa, with at least one species in all analyzed phyla (Fig. 1). In invertebrates, duplicates in the tyrosinase family are present in *N. vectensis* (cnidarians), in *P. fucata* (molluscs), in *C. elegans* (nematodes), in *S. kowalevskii* (hemichordates) and in *B. floridae* (cephalochordates). It is tempting to speculate that the multiple duplicates, present in these organisms, can play diverse functions, besides being involved in melanogenic processes, as phenoloxidases in arthropods that are used also for sclerotization and primary immune response [27], [36], [37]. One can suppose that melanin and its intermediates, in these species, are exploited as a defence mechanism to compensate the lack of a complex and specialized immune system. Notably, amongst all metazoa, amphioxus is the only organism that still retains representative genes of tyr, tyrp and tyr-like groups, firstly described in this paper. The multiple tyrosinases present in *B. floridae* can thus be framed in this perspective, since amphioxus has a simple humoral immune system with lymphocyte-like cells in gills [38], but without clearly identified free, circulating blood cells [39], [40]. Conversely, the absence of tyr-like proteins in *Ciona* could be related to the presence, in this organism, of a well-developed vascular system, in which defined cell types, such as granular amoebocytes and granulocytes, are known to be involved in immunity reactions [39].

Our study confirmed also that the tyrps represent an invention of chordate lineage (Fig. 1A) where they contribute to a more complex melanogenic process. Concerning the topology of the phylogenetic tree for tyrps, we suppose that a rapid evolution, during their appearance in chordates, make it difficult to establish their exact phylogenetic relationship. It seems clear that the duplication events, leading to two copies of tyrps, happened independently in *C. intestinalis*, amphioxus and vertebrates, and that the two copies of vertebrate tyrps derive from a whole genomic duplication event that took place at the base of this lineage.

Taking all this into consideration, we then studied synteny conservation, surrounding the *tyrps* genes in *Ciona* and humans, in order to establish their orthology (Fig. 2). We excluded amphioxus from this analysis because of the short length of the scaffolds. The data indicated a clear orthology between *Ci-tyrp1/2a* and human *TYRP1*, while the absence of shared genes around *Ci-tyrp1/2b* and human *TYRP2* (Fig. 2) did not permit to infer or exclude any orthology.

From the enzymatic point of view, vertebrate tyrp2 is a DOPAchrome tautomerase, responsible for the conversion of L-DOPAchrome into DHICA [41], while the enzymatic role played by tyrp1 is still controversial, since tyrp1 has been attributed either tyrosinase, as well DHICA oxidase, or DOPAchrome tautomerase function but with a low specific activity [42], [43], [44], [45]. Moreover biochemical data indicate that the two vertebrate tyrps, besides acting as enzyme, play also important functions in modulating tyrosinase activity, in the assembly of the melanogenic apparatus and in the detoxification processes taking place within melanosomes [7], [9], [46], [47], [48]. The invention of tyrps could then be related to the need of accessory enzymatic activities to finely tune the melanogenic process. In addition the tyrps could function as structural elements by providing a robust scaffold able to shield tyrosinase from potentially toxic intermediate products of the melanogenic pathway and permit the synthesis of melanin.

### Expression profile and transcriptional regulation of *tyr* and *tyrps* in *Ciona*

Based on the expression profiles, in *Ciona*, *Ci-tyrp1/2a* appears first, at the late gastrula stage, and is copiously expressed up to the tadpole stage in the pigment cell lineage. This could be in agreement with a need to accumulate a large amount of tyrp protein in order to exert its protective and scaffolding function toward tyrosinase in the melanogenic complex. A further interesting feature, that ascidians share with vertebrates, is that *tyrosinase* mRNA (Fig. 3) (and, in *Ciona*, also the corresponding protein product [49]) appears well before pigment synthesis begins [49], [50]. This could be related to the need of a large quantity of enzyme requested at the time of initial melanin synthesis, in order to catalyze the production of sufficient L-DOPA cofactor to maintain a rapid and sustained tyrosine oxidase activity. Interestingly, the expression territories and the timing of *H. roretzy tyrosinase* messenger RNA overlap those of *Ci-tyr* [51]. *H. roretzy tyrp* (*Hr-tyrp*) messenger RNA instead appears earlier (at 110 cell stage) than *Ci-tyrp1/2a*, being localized in blastomeres other than pigment cell lineage [52]. The functional significance of this early expression is not known, but it is important to note that *Hr-tyrp* messenger RNA becomes exclusive of pigment cell lineage from neurula stage onward.

In amphioxus *tyr*, *tyrp1/2a* and *tyrp1/2b* are coexpressed, throughout the epidermal ectoderm, in gastrula and neurula stages and eventually become localized in the rudiment of the primary pigment spot of Hesse organ located in the amphioxus neural tube [53]. The Hesse ocelli represents a characteristic amphioxus trait, not observed in vertebrates, consisting in the presence of bicellular photoreceptor organs (one receptor and one pigment cell), distributed throughout most of the spinal cord and numbered in hundreds in mature animals [54]. Besides Hesse ocelli, amphioxus has another pigment structure, the frontal eye, that differentiates early in the larva and is implicated in controlling orientation to light [54]. The finding that the first spot of Hesse ocelli coexpresses *tyr*, *tyrp1/2a* and *tyrp1/2b* [53] indicates that this pigment cell lineage utilize the same input, as vertebrates, at least for the first pigment spot melanization. Further *in situ* experiments will be instrumental to clarify potential involvement of tyr, tyrp1/2a and tyrp1/2b in melanogenic processes of the frontal eye pigment cell and of all the ocelli that develop in the mature animals. Furthermore, in-depth examination of the expression pattern of the supernumerary *tyr-like* genes, at different developmental stages, will enormously contribute to our understanding of how and when these genes are used in the melanogenic processes in amphioxus.

In the present study we also identified the regulatory regions responsible for the spatio-temporal expression of the three *tyrosinase* family members in *C. intestinalis* (Fig. 4). The ascidian genome is compact, compared with vertebrate genomes, and intergenic regions as well as introns are relatively small [22]. In the case of the three *tyrosinase* family genes, the upstream intergenic regions we have analyzed range between 0.8 and 1.5 kb, which is small enough to easily isolate and clone the whole intergenic region in a reporter expression vector. Similar to transcript levels, the *pCi-tyrp1/2a* enhancer revealed to be the strongest element in driving a robust reporter expression, from the late gastrula stage up to the tadpole stage, in all pigment cell lineage descendants. *pCi-tyrp1/2b* and *pCi-tyr*, instead, although specific, appeared weaker in terms of the number of embryos showing transgene expression. It is likely that these regulatory regions contains elements necessary, but not sufficient, for a robust activation in the endogenous territories. Probably other elements located outside the area we have tested, maybe in the introns, may fill this gap. Thus we have identified relatively short upstream intergenic regions, which are lineage specific and are capable to switch on their activity in a concerted way and at precise developmental times of pigment cell formation. In *C. intestinalis*, lineage restricted enhancers are often used as tool for targeted interference with lineage restricted developmental genes [55], [56], [57]. Our enhancers, given their specificity in labelling the pigment cell lineage, with *pCi-tyrp1/2a* being active from late gastrula and *pCi-tyr* from neurula stages, have been already successfully exploited to interfere with the function of two factors involved in pigment cell differentiation at two consecutive developmental stages [21]. On the other hand, these promoters can give important clues to the network controlling pigment cell development, since tyrosinase family members are typical markers of this lineage.

The bioinformatic analyses we have conducted on these promoters revealed that *C. intestinalis* and *C. savignyi tyrp1/2a* genes share, in their promoter regions, some motifs specific for Pax, Oct/Pou, and Sox family transcription factor bindings (Fig. 5). It is intriguing to note that in *C. intestinalis* the density of specific binding sites in promoter fragments, coupled with their conservation in the corresponding regions of *C. savignyi*, is often indicative of their functional relevance. The distribution of this motif sites is also partially conserved in *Hr-tyrp* promoter. Interestingly, these specific DNA motifs and the corresponding transcription factors have been already demonstrated to be involved in the activation of tyr family members in different vertebrate species [34], thus indicating a certain grade of conservation, among chordates, in the molecular mechanisms controlling pigmentation machinery. Our analysis did not reveal any canonical binding motif for Mitf, a factor fundamental in the development of vertebrate melanin producing cells, both in *C. intestinalis* or *C. savignyi tyr* and *tyrps* 5′ regulatory regions, paralleling the previously findings on *H. roretzi tyr* and *tyrp* promoters [35], [58]. In *Halocynthia*, *Hr-Mitf* messenger RNA becomes localized in the pigment cell lineage from neurula stage onward, as in *C. intestinalis* (http://www.aniseed.cnrs.fr/), and *Hr-Mitf* overexpression is able to activate ectopically *Hr-tyr* [59]. Because canonical Mitf binding sites are absent on ascidian *tyr* and *tyrps* promoters it is possible that Mitf action is mediated through additional transcription factors. Alternatively, we can suppose that ascidian Mitf factors are able to recognize and interact with a non-canonical E-box binding motif.

Collectively the data we have presented here revealed that *tyrosinase* gene family phylogeny is much more painted than previously thought, thus opening new perspectives on the way by which the organisms synthesize and exploit melanin during evolution. Additional expansion of the comparative analysis of *tyr* and *tyrps*, by exploiting the “daily” new sequenced genomes, combined with experiments of *in situ* hybridization and studies on transcriptional regulation of *tyr* and *tyrps* in different phyla, will be crucial for accessing further aspects of pigment cell biology and revealing important mechanisms about their evolution.

## Materials and Methods

### Phylogenetic analysis

Sequences used in phylogenetic analysis were retrieved from the NCBI database and are listed in Table S1. The protein set was aligned using ClustalW [60], [61] and Mega5 [62] with default parameters and the amphioxus tyr protein was taken as reference from its Q19 to the E458 residual aminoacid to set the length of the datasets, in order to avoid regions of unreliable alignment along the extremities of the molecule. *Suberites domuncula* tyr-like was used as outgroup.

Phylogenetic tree reconstructions were carried out using the bayesian (MrBayes), neighbor joining (NJ) and the maximum likelihood (ML) methods. Bayesian trees were inferred using MrBayes 3.1.2 [63], [64]. Two independent runs of 1 million generations each were performed, each with four chains. For convention, convergence was reached when the value for the standard deviation of split frequencies stayed <0.01. NJ and ML analyses were performed using Mega5 [62] and robustness of the obtained tree topologies was assessed with 1000 Bootstrap replicates.

All phylogenetic methods gave similar tree topology, Fig. 1 shows the consensus tree in which each node reports the bootstrap value for MrBayes, ML and NJ respectively.

### Synteny conservation analysis

We analyzed the presence of synteny conservation using the Synteny Database developed by Catchen et al. [65] in addition to manual searches in amphioxus (*B. floridae*, JGI v2.0), *Ciona* (*C. intestinalis*, JGI v2.0) and human (*H. sapiens*, Ensembl release 64) genomes. In Fig. 2 we only show a few representative genes among many that are conserved around the *tyrp* locus. *Ciona* chromosome 5 and human chromosome 9 share an high degree of conservation in the genomic neighborhoods surrounding the *Ci-tryp1/2a* and *Hr-Tyrp1*. Amphioxus *tyrp1/2a* and *tyrp1/2b* lay on scaffold Bf_V2_61 that is most probably not long enough to establish synteny.

### Animals and embryos

Adult *C. intestinalis* were collected from the Gulf of Naples. Animal handling and transgenesis *via* electroporation have been carried out as previously described [66], [67]. Embryo imaging capture was made with a Zeiss Axio Imager M1.

### Whole-mount *in situ* hybridization

Three cDNA clones, presumably encoding tyrosinase family members, were found in *Ciona* genomic database (http://genome.jgi-psf.org/Cioin2/Cioin2.home.html): citb41l04 (N. Satoh Gene Collection 1 ID:CiGC33c19), citb030d10 (N. Satoh Gene Collection 1 ID: CiGC44b23) and cilv069a04 (N. Satoh Gene Collection 1 ID: CiGC31h05). They have been named respectively *Ci-tyr*, *Ci-tyrp1/2a* (previously named *Ci-tyrp1a* [21]) and *Ci-tyrp1/2b*. The corresponding RNA probes were used for whole-mount *in situ* hybridization experiments, performed as previously described [67].

### Construct preparation

*pCi-tyr>mChe* and *pCi-tyrp1/2a>mChe* (originally named *ptyrp1a*>mChe) constructs were previously prepared [21]. Approximately 1.5 kb of the *Ci-tyrp1/2b* 5′ flanking region was PCR-amplified from genomic DNA using the primers: *Ci-ptyrp1/2bF* (GTAGTATAAACAAACTACCGATAACCTGC) and *Ci-ptyrp1/2bR* (AGAACGAAGAAATAGATGTATGCTTGG). The fragment *pCi-tyrp1/2b* was cloned into pCR®II vector (TOPO® TA Cloning Dual Promoter Kit, Invitrogen), following the manufacturer's indications, and then excised through digestion with the unique restriction sites in pCR®II plasmid polylinker (*Hind*III-*Not*I for cloning upstream of mCherry). The digested fragment replaced *pCi-tyr* in *pCi-tyr>mChe* vector (previously digested with *Hind*III-*Not*I to eliminate *ptyr*) to create the construct *pCi-tyrp1/2b>mChe*.

*pCi-tyrp1/2a-0.6>mChe*: the construct *pCi-tyrp1/2a->mChe* was digested with compatible ends generating enzymes, *Spe*I-*Xba*I, to eliminate a fragment of about 0.9 kb at the 5′ end of *pCi-tyrp1a*; the resulting linearized vector was then re-ligated.

### *In silico* analysis for putative *trans*-acting factors

*pCi-tyrp1/2a-0.6*, the corresponding region of *pCs-tyrp1/2a* and *Hr-tyrp-333N* were analyzed using PROMO program (http://alggen.lsi.upc.es/cgi-bin/promo_v3/promo/promoinit.cgi?dirDB=TF_8.3). Only dissimilarity values ≤2% (divergence percentage of the given sequence from the consensus matrix) have been taken into consideration.

## Supporting Information

Figure S1 **Chromosomal distribution of the expanded tyrosinases in** ***B. floridae*** **,** ***S. kowalevskii*** **,** ***N. vectensis*** **and** ***C. elegans*** **. The tandem duplicates lying on the same scaffold or chromosome are** highlighted using the same color code.
(PDF)

Figure S2 **Schematic representation of two metal binding domains (MeA and MeB) and sequence consensus.** A) Key aminoacid positions, probably involved in the change of affinity to phenolic substrates, are reported in pink for tyrosinases and yellow for tyrosinase-related proteins. ø indicates aromatic residues (F, Y or W), x indicates any aminoacid. B) Multiple sequence alignment was obtained using Mega5 software. Conserved residues in all proteins analyzed, highlighted in green, define a robust sequence consensus. Aminoacid changes between tyr and tyrps are represented in pink and yellow, respectively.
(PDF)

Figure S3 **Cysteine residues conservation in Tyrosinase family proteins.** Cysteine (Cys) residues number and positions are reported from deuterostome and protostome organisms. A significant conservation is detectable in deuterostomes; protostomes share with deuterostomes only the N-terminal cluster and present an additional stretch of cysteine residues at the C-terminal.
(PDF)

Figure S4 ***In vivo*** **analysis of** ***pCi-tyrp1/2a-0.6*** **>mChe construct.** Transgenesis was performed *via* electroporation experiments. Merged bright-field/fluorescent images of mCherry expression driven by *pCi-tyrp1/2a.0-6* region at early (A) and middle (B) larval stages (lateral view, anterior is on the left). Note that the transgene expression in pigment cell lineage corresponds to *pCi-tyrp1/2a* full-length enhancer (compare with Fig. 4D).
(PDF)

Table S1 **Accession number of sequences used in the phylogenetic tree of** **Figure 1** **.**
(DOC)
