# Supplementary material for: New Insights into the Evolution of Metazoan Tyrosinase Gene Family
Source: PLoS One. 2012 Apr 20;7(4):e35731. doi: 10.1371/journal.pone.0035731 (PMC3334994; doi:10.1371/journal.pone.0035731)
Supplement: Figure S1 — Chromosomal distribution of the expanded tyrosinases in B. floridae , S. kowalevskii , N. vectensis and C. elegans . The tandem duplicates lying on the same scaffold or chromosome are highlighted using the same color code. (PDF) [file pone.0035731.s001.pdf]

Species	Gene	Genome Locus	NCBI ID	Scaffold or Chromosome	strand
B. floridae	Tyr	BRAFDRAFT_57224	NW_003101456	BRAFLscaffold_149	(–)
	Tyr_a	BRAFDRAFT_22469	NW_003101365	BRAFLscaffold_240	(+)
	Tyr_b	BRAFDRAFT_146037	NW_003101325	BRAFLscaffold_280	(–)
	Tyr_c	BRAFDRAFT_247919	NW_003101369	BRAFLscaffold_236	(+)
	Tyr_d	BRAFDRAFT_219832	NW_003101448	BRAFLscaffold_157	(–)
	Tyr_e	BRAFDRAFT_69010	NW_003101405	BRAFLscaffold_200	(+)
	Tyr_f	BRAFDRAFT_126757	NW_003101510	BRAFLscaffold_095	(–)
S. kowalevskii	Tyr_a	LOC100368593	NW_003140813	Skow_1.1scaffold35909	(+)
	Tyr_b	LOC100376573	NW_003126887	Skow_1.1scaffold21911	(+)
	Tyr_c	LOC100368747	NW_003140813	Skow_1.1scaffold35909	(–)
	Tyr_d	LOC100376875	NW_003126887	Skow_1.1scaffold21911	(+)
N. vectensis	Tyr_a	NEMVEDRAFT_v1g240046	NW_001834391	NEMVEscaffold_23	(–)
	Tyr_b	NEMVEDRAFT_v1g216737	NW_001834144	NEMVEscaffold_270	(+)
	Tyr_c	NEMVEDRAFT_v1g203775	NW_001834369	NEMVEscaffold_45	(–)
	Tyr_d	NEMVEDRAFT_v1g198393	NW_001834405	NEMVEscaffold_9	(+)
C. elegans	Tyr_1	WormBase:WBGene00015332	NC_003281.8	Chromosome III	(+)
	Tyr_2	WormBase:WBGene00010661	NC_003281.8	Chromosome III	(+)
	Tyr_3	WormBase:WBGene00009001	NC_003279.6	Chromosome I	(–)
	Tyr_4	WormBase:WBGene00016419	NC_003279.6	Chromosome I	(+)
