# Supplementary material for: New Insights into the Evolution of Metazoan Tyrosinase Gene Family
Source: PLoS One. 2012 Apr 20;7(4):e35731. doi: 10.1371/journal.pone.0035731 (PMC3334994; doi:10.1371/journal.pone.0035731)
Supplement: Figure S2 — Schematic representation of two metal binding domains (MeA and MeB) and sequence consensus. A) Key aminoacid positions, probably involved in the change of affinity to phenolic substrates, are reported in pink for tyrosinases and yellow for tyrosinase-related proteins. ø indicates aromatic residues (F, Y or W), x indicates any aminoacid. B) Multiple sequence alignment was obtained using Mega5 software. Conserved residues in all proteins analyzed, highlighted in green, define a robust sequence consensus. Aminoacid changes between tyr and tyrps are represented in pink and yellow, respectively. (PDF) [file pone.0035731.s002.pdf]

A

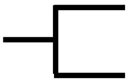

	MeA	MeB
tyr	FxxWHRxF/YLxxWE	DPxFxxHHxxV/I/LDxV/I/LØE/DxW
tyrp	FxxWHRxHLxxLE	DPxFxxLHxxTDxV/I/LØE/DxW

B

	MeA	MeB
**DEUTEROSTOMES**		
1. Branchiostoma floridae tyr_a	FPTWHRAFLQEAE	DPIFLLHHANVDRLFETW
2. Branchiostoma floridae tyr_b	FPTWHRAYVLEIE	DPTFFLHHCNVDRLLETW
3. Branchiostoma floridae tyr_c	FLTWHRAYVLEVE	DPMFFLHHCNVDRLLETW
4. Branchiostoma floridae tyr_d	FPTWHRAFLLDLE	DPIFFLHHCFVDRIFEKW
5. Branchiostoma floridae tyr_e	FLTWHRGYLLEME	DPIFWLHHAFIDRIFEKW
6. Branchiostoma floridae tyr_f	FATWNRAFLVAFE	DPIFYLHRCFMDRIFEKW
7. Branchiostoma floridae tyr	FLPWHRLFLLLWE	DPIFIIHHAYVDSIYEVW
8. Branchiostoma floridae tyrp1/2a	YNTWHRMHLLWLE	DPIFVLLHSYSDAIFDAW
9. Branchiostoma floridae tyrp1/2b	YNTWHRMHLLWLE	DPIFVLLHSYSDAIFDAW
10. Ciona intestinalis tyr	FLPWHRYYLLKWE	DPIFVLHHAYVDSLYELW
11. Ciona intestinalis tyrp1/2a	FLTWHRMHLLGLE	DPIFVLLHTFTDAIFEEW
12. Ciona intestinalis tyrp1/2b	FLTWHRMHLMGLE	DPVFVLLHTFTDAIFDEW
13. Ciona savignyi tyr	FLPWHRYFLLKWE	DPIFLLHHAFVDSIYELW
14. Ciona savignyi tyrp1/2a	FLTWHRMHLLGLE	DPIFVLIHTFTDAIFEEW
15. Ciona savignyi tyrp1/2b	FLTWHRMHLFGLE	DPIFVLLHTFTDAIFEEW
16. Danio rerio tyr	FLPWHRVYLLFWE	DPIFIIHHAFIDSIFEQW
17. Danio rerio tyrp1a	FLTWHRYHLLQLE	DPIFLLLHTFTDAIFDEW
18. Danio rerio tyrp1b	FVTWHRYHLLQLE	DPIFVLLHTFTDAIFDEW
19. Danio rerio tyrp2	FITWHRYHLLSLE	DPIFLVLHAFTDAIFDEW
20. Gallus gallus tyr	FLPWHRAFLLLWE	DPIFILHHAFVDSIFERW
21. Gallus gallus tyrp1	FVTWHRYHLLQLE	DPIFVLLHTFTDAVFDEW
22. Gallus gallus tyrp2	FVTWHRYHLLLLE	DPIFVVLHSFTDAIFDEW
23. Halocynthia roretzi tyr	FLPWHRYFLLKWE	DPLFLVHHVFIDSIYERW
24. Halocynthia roretzi tyrp	FLTWHRMHLLNLE	DPIFVLLHAFTDAIFEEW
25. Homo sapiens TYR	FLPWHRLFLLRWE	DPIFLLHHAFVDSIFEQW
26. Homo sapiens TYRP1	FLTWHRYHLLRLE	DPIFVLLHTFTDAVFDEW
27. Homo sapiens TYRP2	FVTWHRYHLLCLE	DPIFVVIHSFTDAIFDEW
28. Mus musculus tyr	FLPWHRLFLLLWE	DPIFLLHHAFVDSIFEQW
29. Mus musculus tyrp1	FLTWHRYHLLQLE	DPIFVLLHTFTDAVFDEW
30. Mus musculus tyrp2	FVTWHRYHLLWLE	DPVFVVLHSFTDAIFDEW
31. Saccoglossus kowalevskii tyr_a	FPTWHRYFMLLWE	DPIFILHHSNVDRIFEKW
32. Saccoglossus kowalevskii tyr_b	FLTWHRAYLLLFE	DPMFWLHHSNVDRIMEKW
33. Saccoglossus kowalevskii tyr_c	FPTWHRSFIQLWE	DPIFILHHVNVDRILEKW
34. Saccoglossus kowalevskii tyr_d	FLTWHRAYLLLFE	DPMFWLHHSNVDRIMEKW
35. Xenopus tropicalis tyr	FVPWHRYFLLHWE	DPVFVLHHAFVDSIFEQW
36. Xenopus tropicalis tyrp1	FLTWHRYHLMQLE	DPIFVLLHTFTDAVFDEW
37. Xenopus tropicalis tyrp2	FVTWHRYHLLLLE	DPIFVVLHSFTDAIFDEW
**PROTOSTOMES**		
38. Caenorhabditis elegans tyr_a	FLVWHREYMKRME	DPVFFLHHTFVDFIWEMW
39. Caenorhabditis elegans tyr_b	FLPWHREYLKRVE	EPIFFMHHSFVDYLWELW
40. Caenorhabditis elegans tyr_c	FLPWHREFVKRME	DPIFWMHHSFVDLLWEMY
41. Caenorhabditis elegans tyr_d	FLPWHREFVKRVE	DPSFFLHHAFIDFVWEEW
42. Illex argentinus tyr	FLPWHRIYMMIWE	DPAFMLIHGYVDYIWYRF
43. Pinctada fucata tyr_a	FLGWHREYLIMYE	DPVFFFHHTFIDYGWELF
44. Pinctada fucata tyr_b	FLGWHRVYLNMYE	DPVFWFHHLYIDYVWELF
45. Pinctada fucata tyr_c	ILGWHRVFLYLFE	DPLFYFHHCYIDYVWQLF
46. Sepia officinalis tyr	FLPWHRIYMMIWE	DPAFMLIHGYVDYIWYRF
**RADIATA**		
47. Hydra magnipapillata tyr	FYPWHRSYILKFE	SPEFWSHHAMLDGLWFEW
48. Nematostella vectensis tyr_a	FFPWHRWYLLLFE	APEFWFHHGYIDKVWSDW
49. Nematostella vectensis tyr_b	FFPWHRWFILNFE	APEFWFHHGFLDKLWSDW
50. Nematostella vectensis tyr_c	FFPWHRWYLSKIE	TPEFPLHHAFIDKIWDMW
51. Nematostella vectensis tyr_d	FLPWHRQFLLNLE	SPEFLLITAYIDKLWGDW
**PORIFERA**		
52. Suberites domuncula tyr-like	FPVWHRRYMLTVE	DPIFNLHHCNVDRIFESW
