# Supplementary material for: New Insights into the Evolution of Metazoan Tyrosinase Gene Family
Source: PLoS One. 2012 Apr 20;7(4):e35731. doi: 10.1371/journal.pone.0035731 (PMC3334994; doi:10.1371/journal.pone.0035731)
Supplement: Figure S3 — Cysteine residues conservation in Tyrosinase family proteins. Cysteine (Cys) residues number and positions are reported from deuterostome and protostome organisms. A significant conservation is detectable in deuterostomes; protostomes share with deuterostomes only the N-terminal cluster and present an additional stretch of cysteine residues at the C-terminal. (PDF) [file pone.0035731.s003.pdf]

	N-ter	MeA		MeB	C-ter
**Deuterostomes**					
Vertebrates	11		5		-
Ascidians	11		5		8 (tyr)
Cephalochordates	11		5		-
Hemichordates	11		5		-
**Protostomes**					
Nematodes	6		1		≥23
Molluscs	3		1		13
