# Supplementary figures and images for: New Insights into the Evolution of Metazoan Tyrosinase Gene Family
Source: PLoS One. 2012 Apr 20;7(4):e35731. doi: 10.1371/journal.pone.0035731 (PMC3334994; doi:10.1371/journal.pone.0035731)

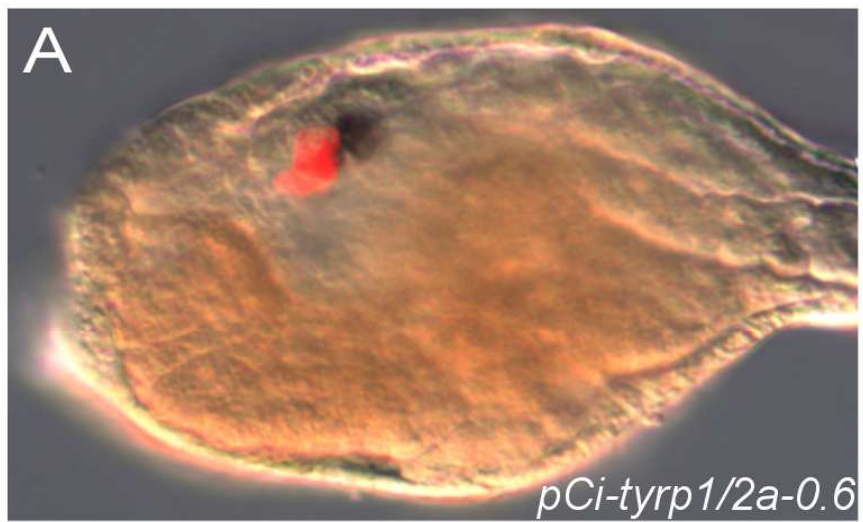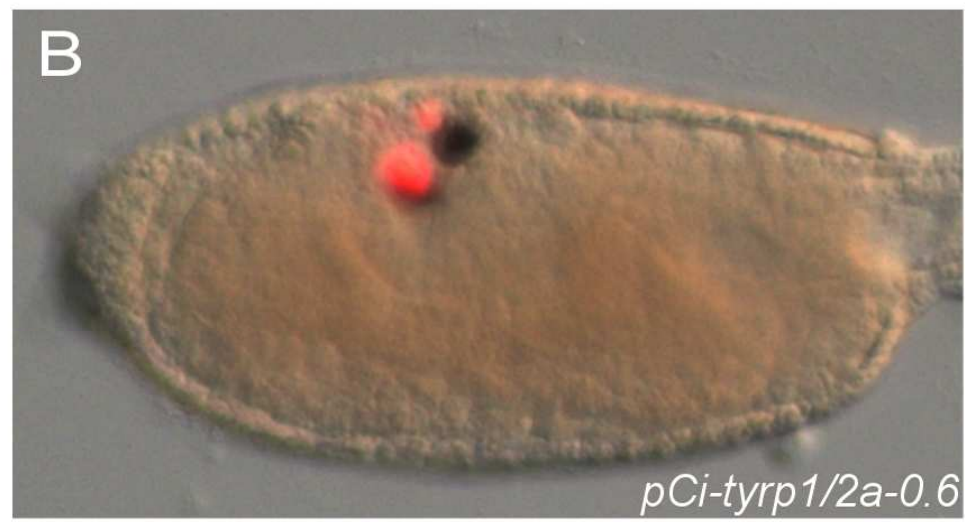

Supplement: Figure S4 — In vivo analysis of pCi-tyrp1/2a-0.6 >mChe construct. Transgenesis was performed via electroporation experiments. Merged bright-field/fluorescent images of mCherry expression driven by pCi-tyrp1/2a.0-6 region at early (A) and middle (B) larval stages (lateral view, anterior is on the left). Note that the transgene expression in pigment cell lineage corresponds to pCi-tyrp1/2a full-length enhancer (compare with Fig. 4D). (PDF) [file pone.0035731.s004.pdf]
