# Supplementary material for: New Insights into the Evolution of Metazoan Tyrosinase Gene Family
Source: PLoS One. 2012 Apr 20;7(4):e35731. doi: 10.1371/journal.pone.0035731 (PMC3334994; doi:10.1371/journal.pone.0035731)
Supplement: Table S1 — Accession number of sequences used in the phylogenetic tree of Figure 1 . (DOC) [file pone.0035731.s005.doc]

Table S1

| **Organism** | **Gene** | **Accession number** |
| --- | --- | --- |
| *Branchiostoma floridae* | tyr | XM_002598584 |
|  | tyrp1/2a | XM_002606855 |
|  | tyrp1/2b | XM_002606856 |
|  | tyr-like_a | XM_002589551 |
|  | tyr-like_b | XM_002587151 |
|  | tyr-like_c | XM_002589786 |
|  | tyr-like_d | XM_002597479 |
|  | tyr-like_e | XM_002592473 |
|  | tyr-like_f | XM_002604545 |
|  |  |  |
| *Caenorhabditis elegans* | Tyra | NM_066310 |
|  | Tyrb | NM_067435 |
|  | Tyrc | NM_059654 |
|  | Tyrd | NM_059308 |
|  |  |  |
| *Ciona intestinalis* | tyr | XM_002123004 |
|  | tyrp1/2a | XM_002119565 |
|  | tyrp1/2b | XM_002129643 |
|  |  |  |
| *Ciona savignyi* | tyr | [ENSCSAVT00000018907](http://www.ensembl.org/Ciona_savignyi/Transcript/Summary?db=core;g=ENSCSAVG00000010989;r=reftig_9:3068829-3075591;t=ENSCSAVT00000018907) |
|  | tyrp1/2a | ENSCSAVT00000002210 |
|  | tyrp1/2b | ENSCSAVT00000009477 |
|  |  |  |
| *Danio rerio* | tyr | NM_131013 |
|  | tyrp1a | BC155086 |
|  | tyrp1b | NM_001002749 |
|  | tyrp2 | NM_131555 |
|  |  |  |
| *Gallus gallus* | tyr | [NM_204160](http://www.ncbi.nlm.nih.gov/nuccore/NM_204160.1) |
|  | tyrp1 | [NM_205045](http://www.ncbi.nlm.nih.gov/nuccore/NM_205045.1) |
|  | tyrp2 | [NM_204935](http://www.ncbi.nlm.nih.gov/nuccore/NM_204935.1) |
|  |  |  |
| *Halocynthia roretzi* | tyr | D63950 |
|  | tyrp | D63949 |
|  |  |  |
| *Homo sapiens* | tyr | [NM_000372](http://www.ncbi.nlm.nih.gov/nuccore/NM_000372.4) |
|  | tyrp1 | [NM_000550](http://www.ncbi.nlm.nih.gov/nuccore/NM_000550.2) |
|  | tyrp2 | [NM_001129889](http://www.ncbi.nlm.nih.gov/nuccore/NM_001129889.1) |
|  |  |  |
| *Hydra magnipapillata* | Tyr | XM_002166300 |
|  |  |  |
| *Illex argentines* | Tyr | AB107880 |
|  |  |  |
| *Mus musculus* | tyr | [NM_011661](http://www.ncbi.nlm.nih.gov/nuccore/NM_011661.4) |
|  | tyrp1 | [NM_031202](http://www.ncbi.nlm.nih.gov/nuccore/NM_031202.2) |
|  | tyrp2 | [NM_010024](http://www.ncbi.nlm.nih.gov/nuccore/NM_010024.3) |
|  |  |  |
| *Nematostella vectensis* | tyr-like_a | XM_001638262 |
|  | tyr-like_b | XM_001625411 |
|  | tyr-like_c | XM_001635746 |
|  | tyr-like_d | XM_001640169 |
|  |  |  |
| *Pinctada fucata* | Tyra | AB254132 |
|  | Tyrb | AB254133 |
|  | Tyrc | DQ112679 |
|  |  |  |
| *Saccoglossus kowalevskii* | Tyra | XM_002738383 |
|  | Tyrb | XM_002735209 |
|  | Tyrc | XM_002738384 |
|  | Tyrd | XM_002735211 |
|  |  |  |
| *Sepia officinalis* | Tyr | AJ297474 |
|  |  |  |
| *Suberites domuncula* | Tyr-like | AJ574915 |
|  |  |  |
| *Xenopus tropicalis* | tyr | NM_001103048 |
|  | tyrp1 | [NM_001016476](http://www.ncbi.nlm.nih.gov/nuccore/NM_001016476.2) |
|  | tyrp2 | NM_001017161 |
